# Supplementary material for: Differences in SARS-CoV-2 antibody prevalence at the end of the pre-vaccination period between age groups: A cross-sectional analysis of the multi-ethnic population-based HELIUS study
Source: PLoS One. 2024 Oct 8;19(10):e0311196. doi: 10.1371/journal.pone.0311196 (PMC11460694; doi:10.1371/journal.pone.0311196)
Supplement: S1 Appendix — (DOCX) [file pone.0311196.s001.docx]

**Supplemental material to ‘Differences in SARS-CoV-2 antibody prevalence at the end of the pre-vaccination period between age groups in a multi-ethnic population: a cross-sectional analysis of the population-based HELIUS study’**

**Table 1**. Distribution of exposure variables among participants of the first two SARS-CoV-2 serological substudy visits (2020-2021), by age groups and ethnic groups (n=2064)

|  | **Younger** | **Middle-aged** | **Older** | **p-value*** |
| --- | --- | --- | --- | --- |
| **Dutch (n=464)** | n=76 | n=119 | n=269 |  |
| Job setting^a^ |  |  |  |  |
| No job/caretaker only | 3 (3.9) | 9 (7.6) | 94 (34.9) | **<0.001** |
| Job with no contact within 1.5 m | 16 (21.1) | 34 (28.6) | 38 (14.1) |  |
| Job with contact within 1.5 m | 57 (75.0) | 76 (63.9) | 137 (50.9) |  |
| Missing | 0 | 0 | 0 |  |
| Occupation level^b^ |  |  |  |  |
| Low | 4 (5.3) | 5 (4.2) | 38 (14.1) | **<0.001** |
| Intermediate | 9 (11.8) | 21 (17.6) | 72 (26.8) |  |
| High | 49 (64.5) | 90 (75.6) | 150 (55.8) |  |
| Missing | 14 (18.4) | 3 (2.5) | 9 (3.3) |  |
| Health literacy^b^ |  |  |  |  |
| Adequate | 76 (100) | 118 (99.2) | 268 (99.6) | 0.67 |
| Low | 0 | 1 (0.8) | 1 (0.4) |  |
| Missing | 0 | 0 | 0 |  |
| Household size^b^ (median, IQR) | 2 (2) | 3 (2) | 2 (1) | **<0.001** |
| Missing | 0 | 0 | 0 |  |
| Household member/steady partner with suspected infection^a^ |  |  |  |  |
| Not applicable | 11 (14.5) | 18 (15.1) | 57 (21.2) | 0.10 |
| No | 52 (68.4) | 82 (68.9) | 189 (70.3) |  |
| Yes | 12 (15.8) | 17 (14.3) | 21 (7.8) |  |
| Missing | 1 (1.3) | 2 (1.7) | 2 (0.7) |  |
| **South-Asian Surinamese (n=391)** | n=61 | n=122 | n=208 |  |
| Job setting^a^ |  |  |  |  |
| No job/caretaker only | 12 (19.7) | 17 (13.9) | 96 (46.2) | **<0.001** |
| Job with no contact within 1.5 m | 8 (13.1) | 17 (13.9) | 28 (13.5) |  |
| Job with contact within 1.5 m | 41 (67.2) | 88 (72.1) | 83 (39.9) |  |
| Missing | 0 | 0 | 1 (0.5) |  |
| Occupation level^b^ |  |  |  |  |
| Low | 19 (31.1) | 32 (26.2) | 88 (42.3) | **<0.01** |
| Intermediate | 16 (26.2) | 48 (39.3) | 65 (31.2) |  |
| High | 14 (23.0) | 35 (28.7) | 33 (15.9) |  |
| Missing | 12 (19.7) | 7 (5.7) | 22 (10.6) |  |
| Health literacy^b^ |  |  |  |  |
| Adequate | 60 (98.4) | 122 (100) | 197 (94.7) | **0.02** |
| Low | 1 (1.6) | 0 | 11 (5.3) |  |
| Missing | 0 | 0 | 0 |  |
| Household size^b^ (median, IQR) | 3 (2) | 3 (2) | 2 (1) | **<0.001** |
| Missing | 0 | 1 (0.8) | 2 (1.0) |  |
| Household member/steady partner with suspected infection^a^ |  |  |  |  |
| Not applicable | 9 (14.8) | 17 (13.9) | 48 (23.1) | **<0.01** |
| No | 41 (67.2) | 92 (75.4) | 149 (71.6) |  |
| Yes | 11 (18.0) | 12 (9.8) | 9 (4.3) |  |
| Missing | 0 | 1 (0.8) | 2 (1.0) |  |
| **African Surinamese (n=360)** | n=30 | n=94 | n=236 |  |
| Job setting^a^ |  |  |  |  |
| No job/caretaker only | 0 | 12 (12.8) | 96 (40.7) | **<0.001** |
| Job with no contact within 1.5 m | 3 (10.0) | 11 (11.7) | 22 (9.3) |  |
| Job with contact within 1.5 m | 27 (90.0) | 71 (75.5) | 118 (50.0) |  |
| Missing | 0 | 0 | 0 |  |
| Occupation level^b^ |  |  |  |  |
| Low | 7 (23.3) | 21 (22.3) | 78 (33.1) | 0.22 |
| Intermediate | 8 (26.7) | 41 (43.6) | 82 (34.7) |  |
| High | 9 (30.0) | 30 (31.9) | 59 (25.0) |  |
| Missing | 6 (20.0) | 2 (2.1) | 17 (7.2) |  |
| Health literacy^b^ |  |  |  |  |
| Adequate | 29 (96.7) | 93 (98.9) | 231 (97.9) | 0.70 |
| Low | 1 (3.3) | 1 (1.1) | 5 (2.1) |  |
| Missing | 0 | 0 | 0 |  |
| Household size^b^ (median, IQR) | 2 (1) | 2 (3) | 2 (2) | 0.43 |
| Missing | 0 | 0 | 5 (2.1) |  |
| Household member/steady partner with suspected infection^a^ |  |  |  |  |
| Not applicable | 7 (23.3) | 15 (16.0) | 70 (29.7) | **0.02** |
| No | 17 (56.7) | 68 (72.3) | 150 (63.6) |  |
| Yes | 6 (20.0) | 11 (11.7) | 16 (6.8) |  |
| Missing | 0 | 0 | 0 |  |
| **Ghanaian (n=213)** | n=20 | n=86 | n=107 |  |
| Job setting^a^ |  |  |  |  |
| No job/caretaker only | 2 (10.0) | 17 (19.8) | 35 (32.7) | 0.10 |
| Job with no contact within 1.5 m | 4 (20.0) | 20 (23.3) | 17 (15.9) |  |
| Job with contact within 1.5 m | 14 (70.0) | 49 (57.0) | 55 (51.4) |  |
| Missing | 0 | 0 | 0 |  |
| Occupation level^b^ |  |  |  |  |
| Low | 16 (80.0) | 59 (68.6) | 77 (72.0) | 0.24 |
| Intermediate | 1 (5.0) | 3 (3.5) | 9 (8.4) |  |
| High | 1 (5.0) | 9 (10.5) | 4 (3.7) |  |
| Missing | 2 (10.0) | 15 (17.4) | 17 (15.9) |  |
| Health literacy^b^ |  |  |  |  |
| Adequate | 17 (85.0) | 52 (60.5) | 67 (62.6) | 0.21 |
| Low | 3 (15.0) | 28 (32.6) | 34 (31.8) |  |
| Missing | 0 | 6 (7.0) | 6 (5.6) |  |
| Household size^b^ (median, IQR) | 4 (2) | 4 (1.3) | 3 (2) | **<0.001** |
| Missing | 1 (5.0) | 6 (7.0) | 6 (5.6) |  |
| Household member/steady partner with suspected infection^a^ |  |  |  |  |
| Not applicable | 0 | 6 (7.0) | 13 (12.1) | **<0.01** |
| No | 16 (80.0) | 75 (87.2) | 93 (86.9) |  |
| Yes | 4 (20.0) | 5 (5.8) | 1 (0.9) |  |
| Missing | 0 | 0 | 0 |  |
| **Turkish (n=324)** | n=78 | n=153 | n=93 |  |
| Job setting^a^ |  |  |  |  |
| No job/caretaker only | 13 (16.7) | 39 (25.5) | 51 (54.8) | **<0.001** |
| Job with no contact within 1.5 m | 16 (20.5) | 26 (17.0) | 9 (9.7) |  |
| Job with contact within 1.5 m | 49 (62.8) | 88 (57.5) | 33 (35.5) |  |
| Missing | 0 | 0 | 0 |  |
| Occupation level^b^ |  |  |  |  |
| Low | 20 (25.6) | 51 (33.3) | 47 (50.5) | **<0.001** |
| Intermediate | 16 (20.5) | 44 (28.8) | 11 (11.8) |  |
| High | 24 (30.8) | 35 (22.9) | 15 (16.1) |  |
| Missing | 18 (23.1) | 23 (15.0) | 20 (21.5) |  |
| Health literacy^b^ |  |  |  |  |
| Adequate | 74 (94.9) | 127 (83.0) | 54 (58.1) | **<0.001** |
| Low | 4 (5.1) | 23 (15.0) | 33 (35.5) |  |
| Missing | 0 | 3 (2.0) | 6 (6.5) |  |
| Household size^b^ (median, IQR) | 3 (2) | 4 (3) | 3 (2) | **0.04** |
| Missing | 1 (1.3) | 2 (1.3) | 7 (7.5) |  |
| Household member/steady partner with suspected infection^a^ |  |  |  |  |
| Not applicable | 12 (15.4) | 19 (12.4) | 14 (15.1) | 0.76 |
| No | 58 (74.4) | 114 (74.5) | 72 (77.4) |  |
| Yes | 8 (10.3) | 19 (12.4) | 7 (7.5) |  |
| Missing | 0 | 1 (0.7) | 0 |  |
| **Moroccan (n=312)** | n=78 | n=144 | n=90 |  |
| Job setting^a^ |  |  |  |  |
| No job/caretaker only | 12 (15.4) | 40 (27.8) | 48 (53.3) | **<0.001** |
| Job with no contact within 1.5 m | 14 (17.9) | 22 (15.3) | 9 (10.0) |  |
| Job with contact within 1.5 m | 52 (66.7) | 80 (55.6) | 33 (36.7) |  |
| Missing | 0 | 2 (1.4) | 0 |  |
| Occupation level^b^ |  |  |  |  |
| Low | 17 (21.8) | 52 (36.1) | 41 (45.6) | **<0.01** |
| Intermediate | 27 (34.6) | 35 (24.3) | 12 (13.3) |  |
| High | 18 (23.1) | 30 (20.8) | 15 (16.7) |  |
| Missing | 16 (20.5) | 27 (18.8) | 22 (24.4) |  |
| Health literacy^b^ |  |  |  |  |
| Adequate | 73 (93.6) | 124 (86.1) | 51 (56.7) | **<0.001** |
| Low | 1 (1.3) | 14 (9.7) | 32 (35.6) |  |
| Missing | 4 (5.1) | 6 (4.2) | 7 (7.8) |  |
| Household size^b^ (median, IQR) | 3 (3) | 4 (3) | 4 (3) | 0.07 |
| Missing | 3 (3.8) | 7 (4.9) | 6 (6.7) |  |
| Household member/steady partner with suspected infection^a^ |  |  |  |  |
| Not applicable | 17 (21.8) | 9 (6.2) | 17 (18.9) | **<0.001** |
| No | 45 (57.7) | 111 (77.1) | 66 (73.3) |  |
| Yes | 16 (20.5) | 21 (14.6) | 7 (7.8) |  |
| Missing | 0 | 3 (2.1) | 0 |  |

Younger: <40 years; middle-aged: 40-54 years; older: ≥55 years.

Data are presented as frequencies with percentages, unless stated otherwise.

Statistically significant p-values (p<0.05) are marked in bold.

* Pearson’s Chi-square tests (for categorical variables) and ANOVA or Kruskal-Wallis tests (for continuous variables)

^a^ Measured at COVID-1 visit.

^b^ Measured at HELIUS baseline measurement.

**Table 2**. Distribution of education level and susceptibility variables among participants of the first two SARS-CoV-2 serological substudy visits (2020-2021), by age groups and ethnic groups (n=2064)

|  | **Younger** | **Middle-aged** | **Older** | **p-value*** |
| --- | --- | --- | --- | --- |
| **Dutch (n=464)** | n=76 | n=119 | n=269 |  |
| Education level^a^ |  |  |  |  |
| Low | 1 (1.3) | 7 (5.9) | 54 (20.1) | **<0.001** |
| Intermediate | 13 (17.1) | 23 (19.3) | 56 (20.8) |  |
| High | 62 (81.6) | 88 (73.9) | 159 (59.1) |  |
| Missing | 0 | 1 (0.8) | 0 |  |
| *Susceptibility variables* |  |  |  |  |
| Vitamin D intake^b^ |  |  |  |  |
| Yes | 35 (46.1) | 60 (50.4) | 170 (63.2) | **<0.01** |
| No | 41 (53.9) | 59 (49.6) | 99 (36.8) |  |
| Missing | 0 | 0 | 0 |  |
| BMI^c^ (mean, SD) | 23.4 (3.2) | 24.3 (3.9) | 26.3 (4.4) | **<0.001** |
| Missing | 1 (1.3) | 0 | 5 (1.9) |  |
| Systolic blood pressure^c^ (mean, SD) | 113.9 (14.2) | 115.9 (12.9) | 128.8 (17.1) | **<0.001** |
| Missing | 1 (1.3) | 0 | 5 (1.9) |  |
| Haemoglobin^c^ (mean, SD) | 8.8 (0.8) | 8.8 (0.7) | 8.9 (0.7) | 0.31 |
| Missing | 2 (2.6) | 2 (1.7) | 8 (3.0) |  |
| Number of comorbidities^c^ |  |  |  |  |
| 0 comorbidities | 66 (86.8) | 93 (78.2) | 112 (41.6) | **<0.001** |
| 1 comorbidity | 6 (7.9) | 23 (19.3) | 105 (39.0) |  |
| ≥2 comorbidities | 3 (3.9) | 3 (2.5) | 47 (17.5) |  |
| Missing | 1 (1.3) | 0 | 5 (1.9) |  |
| **South-Asian Surinamese (n=391)** | n=61 | n=122 | n=208 |  |
| Education level^a^ |  |  |  |  |
| Low | 10 (16.4) | 42 (34.4) | 128 (61.5) | **<0.001** |
| Intermediate | 32 (52.5) | 48 (39.3) | 41 (19.7) |  |
| High | 19 (31.1) | 32 (26.2) | 38 (18.3) |  |
| Missing | 0 | 0 | 1 (0.5) |  |
| *Susceptibility variables* |  |  |  |  |
| Vitamin D intake^b^ |  |  |  |  |
| Yes | 31 (50.8) | 76 (62.3) | 146 (70.2) | **0.01** |
| No | 30 (49.2) | 45 (36.9) | 60 (28.8) |  |
| Missing | 0 | 1 (0.8) | 2 (1.0) |  |
| BMI^c^ (mean, SD) | 25.9 (5.1) | 27.0 (4.7) | 27.5 (4.7) | 0.07 |
| Missing | 2 (3.3) | 1 (0.8) | 0 |  |
| Systolic blood pressure^c^ (mean, SD) | 115.7 (14.3) | 126.8 (19.2) | 132.0 (18.5) | **<0.001** |
| Missing | 4 (6.6) | 1 (0.8) | 0 |  |
| Haemoglobin^c^ (mean, SD) | 8.5 (1.0) | 8.5 (1.0) | 8.5 (0.9) | 0.85 |
| Missing | 2 (3.3) | 2 (1.6) | 5 (2.4) |  |
| Number of comorbidities^c^ |  |  |  |  |
| 0 comorbidities | 47 (77.0) | 63 (51.6) | 50 (24.0) | **<0.001** |
| 1 comorbidity | 10 (16.4) | 36 (29.5) | 71 (34.1) |  |
| ≥2 comorbidities | 2 (3.3) | 22 (18.0) | 87 (41.8) |  |
| Missing | 2 (3.3) | 1 (0.8) | 0 |  |
| **African Surinamese (n=360)** | n=30 | n=94 | n=236 |  |
| Education level^a^ |  |  |  |  |
| Low | 4 (13.3) | 24 (25.5) | 94 (39.8) | **<0.001** |
| Intermediate | 9 (30.0) | 42 (44.7) | 73 (30.9) |  |
| High | 17 (56.7) | 28 (29.8) | 67 (28.4) |  |
| Missing | 0 | 0 | 2 (0.8) |  |
| *Susceptibility variables* |  |  |  |  |
| Vitamin D intake^b^ |  |  |  |  |
| Yes | 14 (46.7) | 47 (50.0) | 139 (58.9) | 0.20 |
| No | 16 (53.3) | 47 (50.0) | 97 (41.1) |  |
| Missing | 0 | 0 | 0 |  |
| BMI^c^ (mean, SD) | 26.1 (5.5) | 27.7 (4.2) | 28.2 (5.1) | 0.10 |
| Missing | 1 (3.3) | 1 (1.1) | 2 (0.8) |  |
| Systolic blood pressure^c^ (mean, SD) | 115.2 (13.7) | 127.0 (17.3) | 135.1 (18.4) | **<0.001** |
| Missing | 1 (3.3) | 2 (2.1) | 2 (0.8) |  |
| Haemoglobin^c^ (mean, SD) | 8.4 (0.9) | 8.4 (1.2) | 8.5 (0.9) | 0.64 |
| Missing | 1 (3.3) | 2 (2.1) | 4 (1.7) |  |
| Number of comorbidities^c^ |  |  |  |  |
| 0 comorbidities | 23 (76.7) | 55 (58.5) | 47 (19.9) | **<0.001** |
| 1 comorbidity | 6 (20.0) | 33 (35.1) | 108 (45.8) |  |
| ≥2 comorbidities | 0 | 5 (5.3) | 79 (33.5) |  |
| Missing | 1 (3.3) | 1 (1.1) | 2 (0.8) |  |
| **Ghanaian (n=213)** | n=20 | n=86 | n=107 |  |
| Education level^a^ |  |  |  |  |
| Low | 12 (60.0) | 55 (64.0) | 67 (62.6) | 0.54 |
| Intermediate | 5 (25.0) | 15 (17.4) | 26 (24.3) |  |
| High | 3 (15.0) | 10 (11.6) | 7 (6.5) |  |
| Missing | 0 | 6 (7.0) | 7 (6.5) |  |
| *Susceptibility variables* |  |  |  |  |
| Vitamin D intake^b^ |  |  |  |  |
| Yes | 12 (60.0) | 45 (52.3) | 53 (49.5) | 0.68 |
| No | 8 (40.0) | 41 (47.7) | 54 (50.5) |  |
| Missing | 0 | 0 | 0 |  |
| BMI^c^ (mean, SD) | 28.4 (6.1) | 29.0 (3.7) | 29.3 (4.5) | 0.71 |
| Missing | 1 (5.0) | 0 | 2 (1.9) |  |
| Systolic blood pressure^c^ (mean, SD) | 124.4 (20.1) | 137.2 (19.4) | 138.2 (16.3) | **<0.01** |
| Missing | 1 (5.0) | 0 | 2 (1.9) |  |
| Haemoglobin^c^ (mean, SD) | 8.1 (1.2) | 8.3 (1.0) | 8.6 (0.9) | **0.03** |
| Missing | 1 (5.0) | 1 (1.2) | 2 (1.9) |  |
| Number of comorbidities^c^ |  |  |  |  |
| 0 comorbidities | 13 (65.0) | 32 (37.2) | 19 (17.8) | **<0.001** |
| 1 comorbidity | 6 (30.0) | 39 (45.3) | 52 (48.6) |  |
| ≥2 comorbidities | 0 | 15 (17.4) | 34 (31.8) |  |
| Missing | 1 (5.0) | 0 | 2 (1.9) |  |
| **Turkish (n=324)** | n=78 | n=153 | n=93 |  |
| Education level^a^ |  |  |  |  |
| Low | 14 (17.9) | 57 (37.3) | 49 (52.7) | **<0.001** |
| Intermediate | 33 (42.3) | 50 (32.7) | 15 (16.1) |  |
| High | 30 (38.5) | 44 (28.8) | 21 (22.6) |  |
| Missing | 1 (1.3) | 2 (1.3) | 8 (8.6) |  |
| *Susceptibility variables* |  |  |  |  |
| Vitamin D intake^b^ |  |  |  |  |
| Yes | 31 (39.7) | 81 (52.9) | 46 (49.5) | 0.15 |
| No | 47 (60.3) | 71 (46.4) | 47 (50.5) |  |
| Missing | 0 | 1 (0.7) | 0 |  |
| BMI^c^ (mean, SD) | 26.2 (5.0) | 28.4 (5.0) | 30.2 (5.7) | **<0.001** |
| Missing | 2 (2.6) | 2 (1.3) | 0 |  |
| Systolic blood pressure^c^ (mean, SD) | 114.8 (12.8) | 118.4 (16.2) | 130.7 (18.9) | **<0.001** |
| Missing | 2 (2.6) | 2 (1.3) | 0 |  |
| Haemoglobin^c^ (mean, SD) | 8.8 (1.1) | 8.6 (1.0) | 8.8 (0.7) | 0.15 |
| Missing | 2 (2.6) | 2 (1.3) | 0 |  |
| Number of comorbidities^c^ |  |  |  |  |
| 0 comorbidities | 69 (88.5) | 93 (60.8) | 35 (37.6) | **<0.001** |
| 1 comorbidity | 7 (9.0) | 45 (29.4) | 30 (32.3) |  |
| ≥2 comorbidities | 0 | 13 (8.5) | 28 (30.1) |  |
| Missing | 2 (2.6) | 2 (1.3) | 0 |  |
| **Moroccan (n=312)** | n=78 | n=144 | n=90 |  |
| Education level^a^ |  |  |  |  |
| Low | 7 (9.0) | 63 (43.8) | 48 (53.3) | **<0.001** |
| Intermediate | 32 (41.0) | 46 (31.9) | 20 (22.2) |  |
| High | 35 (44.9) | 30 (20.8) | 15 (16.7) |  |
| Missing | 4 (5.1) | 5 (3.5) | 7 (7.8) |  |
| *Susceptibility variables* |  |  |  |  |
| Vitamin D intake^b^ |  |  |  |  |
| Yes | 28 (35.9) | 65 (45.1) | 40 (44.4) | 0.38 |
| No | 50 (64.1) | 79 (54.9) | 50 (55.6) |  |
| Missing | 0 | 0 | 0 |  |
| BMI^c^ (mean, SD) | 26.8 (4.6) | 27.9 (3.9) | 29.0 (4.9) | **<0.01** |
| Missing | 1 (1.3) | 2 (1.4) | 1 (1.1) |  |
| Systolic blood pressure^c^ (mean, SD) | 111.1 (11.6) | 121.3 (15.3) | 134.7 (17.2) | **<0.001** |
| Missing | 2 (2.6) | 2 (1.4) | 1 (1.1) |  |
| Haemoglobin^c^ (mean, SD) | 8.5 (1.0) | 8.6 (1.1) | 8.7 (1.0) | 0.62 |
| Missing | 1 (1.3) | 2 (1.4) | 1 (1.1) |  |
| Number of comorbidities^c^ |  |  |  |  |
| 0 comorbidities | 73 (93.6) | 100 (69.4) | 21 (23.3) | **<0.001** |
| 1 comorbidity | 4 (5.1) | 27 (18.8) | 48 (53.3) |  |
| ≥2 comorbidities | 0 | 15 (10.4) | 20 (22.2) |  |
| Missing | 1 (1.3) | 2 (1.4) | 1 (1.1) |  |

Younger: <40 years; middle-aged: 40-54 years; older: ≥55 years.

Data are presented as frequencies with percentages, unless stated otherwise.

Statistically significant p-values (p<0.05) are marked in bold.

Number of comorbidities include hypertension, prior CVD, diabetes, CKD risk, and asthma/COPD.

* Pearson’s Chi-square tests (for categorical variables) and ANOVA or Kruskal-Wallis tests (for continuous variables).

^a^ Measured at baseline.

^b^ Measured at COVID-2 visit.

^c^ Measured at HELIUS follow-up visit (for some participants, the HELIUS follow-up visit and COVID-1 visit were combined, while for others, the HELIUS follow-up visit took place before COVID-1 visit).

**Table 3**. Distribution of individual comorbidities among participants of the first two SARS-CoV-2 serological substudy visits (2020-2021), by age groups and ethnic groups (n=2064)

|  | **Younger** | **Middle-aged** | **Older** | **p-value*** |
| --- | --- | --- | --- | --- |
| **Dutch (n=464)** | n=76 | n=119 | n=269 |  |
| Hypertension |  |  |  |  |
| No | 67 (88.2) | 103 (86.6) | 139 (51.7) | **<0.001** |
| Yes | 8 (10.5) | 16 (13.4) | 125 (46.5) |  |
| Missing | 1 (1.3) | 0 | 5 (1.9) |  |
| Prior CVD |  |  |  |  |
| No | 75 (98.7) | 117 (98.3) | 239 (88.8) | **<0.001** |
| Yes | 0 | 2 (1.7) | 25 (9.3) |  |
| Missing | 1 (1.3) | 0 | 5 (1.9) |  |
| Diabetes |  |  |  |  |
| No | 74 (97.4) | 117 (98.3) | 237 (88.1) | **<0.001** |
| Yes | 0 | 2 (1.7) | 27 (10.0) |  |
| Missing | 2 (2.6) | 0 | 5 (1.9) |  |
| CKD risk |  |  |  |  |
| Low | 36 (47.4) | 91 (76.5) | 157 (58.4) | 0.08 |
| Moderate | 1 (1.3) | 1 (0.8) | 14 (5.2) |  |
| High (including very high) | 0 | 1 (0.8) | 5 (1.9) |  |
| Missing | 39 (51.3) | 26 (21.8) | 93 (34.6) |  |
| Asthma/COPD |  |  |  |  |
| No | 72 (94.7) | 112 (94.1) | 246 (91.4) | 0.66 |
| Yes | 3 (3.9) | 7 (5.9) | 18 (6.7) |  |
| Missing | 1 (1.3) | 0 | 5 (1.9) |  |
| **South-Asian Surinamese (n=391)** | n=61 | n=122 | n=208 |  |
| Hypertension |  |  |  |  |
| No | 49 (80.3) | 73 (59.8) | 67 (32.2) | **<0.001** |
| Yes | 8 (13.1) | 48 (39.3) | 141 (67.8) |  |
| Missing | 4 (6.6) | 1 (0.8) | 0 |  |
| Prior CVD |  |  |  |  |
| No | 57 (93.4) | 113 (92.6) | 183 (88.0) | 0.07 |
| Yes | 2 (3.3) | 8 (6.6) | 25 (12.0) |  |
| Missing | 2 (3.3) | 1 (0.8) | 0 |  |
| Diabetes |  |  |  |  |
| No | 56 (91.8) | 102 (83.6) | 137 (65.9) | **<0.001** |
| Yes | 3 (4.9) | 19 (15.6) | 71 (34.1) |  |
| Missing | 2 (3.3) | 1 (0.8) | 0 |  |
| CKD risk |  |  |  |  |
| Low | 45 (73.8) | 92 (75.4) | 135 (64.9) | **0.02** |
| Moderate | 1 (1.6) | 8 (6.6) | 24 (11.5) |  |
| High (including very high) | 0 | 2 (1.6) | 10 (4.8) |  |
| Missing | 15 (24.6) | 20 (16.4) | 39 (18.8) |  |
| Asthma/COPD |  |  |  |  |
| No | 59 (96.7) | 116 (95.1) | 191 (91.8) | **0.04** |
| Yes | 0 | 5 (4.1) | 17 (8.2) |  |
| Missing | 2 (3.3) | 1 (0.8) | 0 |  |
| **African Surinamese (n=360)** | n=30 | n=94 | n=236 |  |
| Hypertension |  |  |  |  |
| No | 26 (86.7) | 57 (60.6) | 60 (25.4) | **<0.001** |
| Yes | 3 (10.0) | 35 (37.2) | 174 (73.7) |  |
| Missing | 1 (3.3) | 2 (2.1) | 2 (0.8) |  |
| Prior CVD |  |  |  |  |
| No | 29 (96.7) | 91 (96.8) | 207 (87.7) | **<0.01** |
| Yes | 0 | 2 (2.1) | 27 (11.4) |  |
| Missing | 1 (3.3) | 1 (1.1) | 2 (0.8) |  |
| Diabetes |  |  |  |  |
| No | 29 (96.7) | 89 (94.7) | 183 (77.5) | **<0.001** |
| Yes | 0 | 4 (4.3) | 49 (20.8) |  |
| Missing | 1 (3.3) | 1 (1.1) | 4 (1.7) |  |
| CKD risk |  |  |  |  |
| Low | 24 (80.0) | 73 (77.7) | 176 (74.6) | **0.02** |
| Moderate | 1 (3.3) | 1 (1.1) | 23 (9.7) |  |
| High (including very high) | 0 | 0 | 7 (3.0) |  |
| Missing | 5 (16.7) | 20 (21.3) | 30 (12.7) |  |
| Asthma/COPD |  |  |  |  |
| No | 27 (90.0) | 92 (97.9) | 223 (94.5) | 0.22 |
| Yes | 2 (6.7) | 1 (1.1) | 11 (4.7) |  |
| Missing | 1 (3.3) | 1 (1.1) | 2 (0.8) |  |
| **Ghanaian (n=213)** | n=20 | n=86 | n=107 |  |
| Hypertension |  |  |  |  |
| No | 13 (65.0) | 38 (44.2) | 26 (24.3) | **<0.001** |
| Yes | 6 (30.0) | 48 (55.8) | 79 (73.8) |  |
| Missing | 1 (5.0) | 0 | 2 (1.9) |  |
| Prior CVD |  |  |  |  |
| No | 19 (95.0) | 82 (95.3) | 100 (93.5) | 0.63 |
| Yes | 0 | 4 (4.7) | 5 (4.7) |  |
| Missing | 1 (5.0) | 0 | 2 (1.9) |  |
| Diabetes |  |  |  |  |
| No | 19 (95.0) | 77 (89.5) | 80 (74.8) | **<0.01** |
| Yes | 0 | 9 (10.5) | 25 (23.4) |  |
| Missing | 1 (5.0) | 0 | 2 (1.9) |  |
| CKD risk |  |  |  |  |
| Low | 18 (90.0) | 77 (89.5) | 90 (84.1) | 0.39 |
| Moderate | 0 | 9 (10.5) | 9 (8.4) |  |
| High (including very high) | 0 | 0 | 2 (1.9) |  |
| Missing | 2 (10.0) | 0 | 6 (5.6) |  |
| Asthma/COPD |  |  |  |  |
| No | 19 (95.0) | 84 (97.7) | 101 (94.4) | 0.61 |
| Yes | 0 | 2 (2.3) | 4 (3.7) |  |
| Missing | 1 (5.0) | 0 | 2 (1.9) |  |
| **Turkish (n=324)** | n=78 | n=153 | n=93 |  |
| Hypertension |  |  |  |  |
| No | 72 (92.3) | 106 (69.3) | 45 (48.4) | **<0.001** |
| Yes | 4 (5.1) | 45 (29.4) | 48 (51.6) |  |
| Missing | 2 (2.6) | 2 (1.3) | 0 |  |
| Prior CVD |  |  |  |  |
| No | 75 (96.2) | 146 (95.4) | 82 (88.2) | **<0.01** |
| Yes | 1 (1.3) | 5 (3.3) | 11 (11.8) |  |
| Missing | 2 (2.6) | 2 (1.3) | 0 |  |
| Diabetes |  |  |  |  |
| No | 75 (96.2) | 139 (90.8) | 74 (79.6) | **<0.001** |
| Yes | 1 (1.3) | 12 (7.8) | 19 (20.4) |  |
| Missing | 2 (2.6) | 2 (1.3) | 0 |  |
| CKD risk |  |  |  |  |
| Low | 58 (74.4) | 111 (72.5) | 68 (73.1) | **0.04** |
| Moderate | 0 | 7 (4.6) | 8 (8.6) |  |
| High (including very high) | 0 | 0 | 0 |  |
| Missing | 20 (25.6) | 35 (22.9) | 17 (18.3) |  |
| Asthma/COPD |  |  |  |  |
| No | 75 (96.2) | 145 (94.8) | 83 (89.2) | **0.01** |
| Yes | 1 (1.3) | 6 (3.9) | 10 (10.8) |  |
| Missing | 2 (2.6) | 2 (1.3) | 0 |  |
| **Moroccan (n=312)** | n=78 | n=144 | n=90 |  |
| Hypertension |  |  |  |  |
| No | 72 (92.3) | 106 (69.3) | 45 (48.4) | **<0.001** |
| Yes | 4 (5.1) | 45 (29.4) | 48 (51.6) |  |
| Missing | 2 (2.6) | 2 (1.3) | 0 |  |
| Prior CVD |  |  |  |  |
| No | 77 (98.7) | 139 (96.5) | 84 (93.3) | 0.07 |
| Yes | 0 | 3 (2.1) | 5 (5.6) |  |
| Missing | 1 (1.3) | 2 (1.4) | 1 (1.1) |  |
| Diabetes |  |  |  |  |
| No | 77 (98.7) | 133 (92.4) | 63 (70.0) | **<0.001** |
| Yes | 0 | 9 (6.2) | 26 (28.9) |  |
| Missing | 1 (1.3) | 2 (1.4) | 1 (1.1) |  |
| CKD risk |  |  |  |  |
| Low | 42 (53.8) | 91 (63.2) | 71 (78.9) | 0.43 |
| Moderate | 0 | 6 (4.2) | 6 (6.7) |  |
| High (including very high) | 0 | 1 (0.7) | 1 (1.1) |  |
| Missing | 36 (46.2) | 46 (31.9) | 12 (13.3) |  |
| Asthma/COPD |  |  |  |  |
| No | 75 (96.2) | 137 (95.1) | 83 (92.2) | 0.35 |
| Yes | 2 (2.6) | 5 (3.5) | 6 (6.7) |  |
| Missing | 1 (1.3) | 2 (1.4) | 1 (1.1) |  |

Younger: <40 years; middle-aged: 40-54 years; older: ≥55 years.

Data are presented as frequencies with percentages, unless stated otherwise.

Statistically significant p-values (p<0.05) are marked in bold.

All variables were measured at HELIUS follow-up visit.

* Pearson’s Chi-square tests

**Table 4.** Distribution of SARS-CoV-2 antibody test result at the second SARS-CoV-2 serological substudy visit (2020-2021), by age and ethnicity (n=2064)

|  | **Younger** | **Middle-aged** | **Older** | **p-value*** |
| --- | --- | --- | --- | --- |
| **Dutch (n=464)** | n=76 | n=119 | n=269 |  |
| SARS-CoV-2 antibody test result |  |  |  |  |
| Negative | 66 (86.8) | 106 (89.1) | 244 (90.7) | 0.60 |
| Positive | 10 (13.2) | 13 (10.9) | 25 (9.3) |  |
| **South-Asian Surinamese (n=391)** | n=61 | n=122 | n=208 |  |
| SARS-CoV-2 antibody test result |  |  |  |  |
| Negative | 50 (82.0) | 98 (80.3) | 173 (83.2) | 0.81 |
| Positive | 11 (18.0) | 24 (19.7) | 35 (16.8) |  |
| **African Surinamese (n=360)** | n=30 | n=94 | n=236 |  |
| SARS-CoV-2 antibody test result |  |  |  |  |
| Negative | 21 (70.0) | 75 (79.8) | 192 (81.4) | 0.34 |
| Positive | 9 (30.0) | 19 (20.2) | 44 (18.6) |  |
| **Ghanaian (n=213)** | n=20 | n=86 | n=107 |  |
| SARS-CoV-2 antibody test result |  |  |  |  |
| Negative | 12 (60.0) | 41 (47.7) | 56 (52.3) | 0.58 |
| Positive | 8 (40.0) | 45 (52.3) | 51 (47.7) |  |
| **Turkish (n=324)** | n=78 | n=153 | n=93 |  |
| SARS-CoV-2 antibody test result |  |  |  |  |
| Negative | 51 (65.4) | 111 (72.5) | 64 (68.8) | 0.52 |
| Positive | 27 (34.6) | 42 (27.5) | 29 (31.2) |  |
| **Moroccan (n=312)** | n=78 | n=144 | n=90 |  |
| SARS-CoV-2 antibody test result |  |  |  |  |
| Negative | 51 (65.4) | 95 (66.0) | 63 (70.0) | 0.77 |
| Positive | 27 (34.6) | 49 (34.0) | 27 (30.0) |  |

Younger: <40 years; middle-aged: 40-54 years; older: ≥55 years.

Data are presented as frequencies with percentages.

* Pearson’s Chi-square test

**Table 5.** Associations between age and SARS-CoV-2 antibodies, adjusted for education, susceptibility variables, and exposure, by ethnicity

|  | **Prevalence ratios (95% CI) for SARS-CoV-2 infection** | | | | | |
| --- | --- | --- | --- | --- | --- | --- |
|  | **Dutch (n=464)** | **South-Asian Surinamese (n=391)** | **African Surinamese (n=360)** | **Ghanaian (n=213)** | **Turkish (n=324)** | **Moroccan (n=312)** |
| Model 1^a^ |  |  |  |  |  |  |
| Younger | 2.55 (0.93-6.97) | 0.79 (0.26-2.40) | 1.30 (0.49-3.47) | 0.57 (0.27-1.24) | 1.40 (0.60-3.30) | 1.27 (0.61-2.65) |
| Middle-aged | Ref | Ref | Ref | Ref | Ref | Ref |
| Older | 0.53 (0.16-1.74) | 0.82 (0.40-1.68) | 0.78 (0.36-1.69) | 0.95 (0.56-1.60) | 1.53 (0.72-3.26) | 0.89 (0.46-1.70) |
| **Education level** |  |  |  |  |  |  |
| Model 1 + education level |  |  |  |  |  |  |
| Younger | 2.40 (0.88-6.53) | 0.87 (0.28-2.70) | 1.39 (0.55-3.53) | 0.58 (0.26-1.25) | 1.45 (0.61-3.43) | 1.35 (0.63-2.86) |
| Middle-aged | Ref | Ref | Ref | Ref | Ref | Ref |
| Older | 0.49 (0.14-1.73) | 0.73 (0.36-1.50) | 0.88 (0.40-1.94) | 0.94 (0.56-1.60) | 1.51 (0.70-3.26) | 0.84 (0.43-1.64) |
| **Susceptibility variables** |  |  |  |  |  |  |
| Model 1^b^ + vitamin D intake |  |  |  |  |  |  |
| Younger | 2.40 (0.88-6.58) | 0.87 (0.28-2.73) | 1.33 (0.52-3.39) | 0.55 (0.25-1.21) | 1.48 (0.63-3.50) | 1.33 (0.63-2.83) |
| Middle-aged | Ref | Ref | Ref | Ref | Ref | Ref |
| Older | 0.49 (0.14-1.73) | 0.73 (0.36-1.48) | 0.83 (0.37-1.87) | 0.96 (0.56-1.64) | 1.50 (0.70-3.22) | 0.84 (0.43-1.64) |
| Model 1^b^ + BMI |  |  |  |  |  |  |
| Younger | **2.83 (1.03-7.83)** | 0.87 (0.29-2.59) | 1.51 (0.53-4.35) | 0.55 (0.24-1.27) | 1.23 (0.50-3.00) | 1.41 (0.66-3.01) |
| Middle-aged | Ref | Ref | Ref | Ref | Ref | Ref |
| Older | 0.37 (0.11-1.22) | 0.77 (0.37-1.60) | 0.84 (0.38-1.84) | 0.88 (0.52-1.49) | 1.64 (0.74-3.64) | 0.85 (0.43-1.67) |
| Model 1^b^ + systolic blood pressure |  |  |  |  |  |  |
| Younger | 2.57 (0.91-7.23) | 0.95 (0.31-2.98) | 1.45 (0.50-4.19) | 0.55 (0.24-1.26) | 1.22 (0.54-2.78) | 1.42 (0.66-3.07) |
| Middle-aged | Ref | Ref | Ref | Ref | Ref | Ref |
| Older | 0.38 (0.11-1.38) | 0.69 (0.33-1.43) | 0.87 (0.41-1.87) | 0.83 (0.48-1.41) | 1.65 (0.96-2.81) | 0.83 (0.41-1.68) |
| Model 1^b^ + haemoglobin level |  |  |  |  |  |  |
| Younger | **2.79 (1.01-7.72)** | 0.89 (0.29-2.72) | 1.52 (0.52-4.40) | 0.54 (0.24-1.23) | 1.25 (0.51-3.09) | 1.39 (0.64-2.98) |
| Middle-aged | Ref | Ref | Ref | Ref | Ref | Ref |
| Older | 0.47 (0.13-1.72) | 0.72 (0.35-1.50) | 0.87 (0.40-1.87) | 0.93 (0.54-1.58) | 1.57 (0.71-3.49) | 0.87 (0.44-1.70) |
| Model 1^b^ + number of comorbidities |  |  |  |  |  |  |
| Younger | **2.83 (1.04-7.74)** | 0.95 (0.31-2.91) | 1.33 (0.46-3.82) | 0.56 (0.25-1.26) | 1.22 (0.50-3.01) | 1.43 (0.67-3.06) |
| Middle-aged | Ref | Ref | Ref | Ref | Ref | Ref |
| Older | 0.39 (0.11-1.38) | 0.66 (0.32-1.39) | 0.96 (0.43-2.13) | 0.87 (0.52-1.46) | 1.65 (0.74-3.66) | 0.84 (0.41-1.70) |

Younger: <40 years; middle-aged: 40-54 years; older: ≥55 years.

Statistically significant p-values (p<0.05) are marked in bold.

^a^ Model 1 adjustments: age, sex and exposure variables (job setting, occupation level, health literacy, household size, household member with suspected infection). Adjustments for exposure variables differ per ethnic group:

- Dutch: all, but health literacy;
- South-Asian Surinamese: all, but household member with suspected infection;
- Ghanaian: all, but job setting and household size;
- African Surinamese, Turkish, Moroccan: all.

^b^ Additionally adjusted for education level.

**Table 6.** Associations between exposure variables, education level, susceptibility variables and presence of SARS-CoV-2 antibodies (prevalence ratios), per ethnic group

|  | **Prevalence ratios (95% CI) for SARS-CoV-2 infection** | | | | | |
| --- | --- | --- | --- | --- | --- | --- |
|  | **Dutch (n=464)** | **South-Asian Surinamese (n=391)** | **African Surinamese (n=360)** | **Ghanaian (n=213)** | **Turkish (n=324)** | **Moroccan (n=312)** |
| **Exposure variables** |  |  |  |  |  |  |
| Job setting |  |  |  |  |  |  |
| No job/caretaker only | Ref | Ref | Ref | Ref | Ref | Ref |
| Job with no contact within 1.5 m | 0.94 (0.34-2.59) | 0.80 (0.37-1.75) | 0.46 (0.14-1.55) | 0.99 (0.63-1.57) | 1.06 (0.64-1.74) | 1.08 (0.66-1.78) |
| Job with contact within 1.5 m | 1.34 (0.63-2.87) | 1.20 (0.73-1.98) | 1.31 (0.73-2.35) | 1.16 (0.82-1.64) | 0.91 (0.61-1.35) | 0.85 (0.58-1.24) |
| Occupation level |  |  |  |  |  |  |
| Low | 0.60 (0.19-1.88) | 1.72 (0.89-3.34) | 1.59 (0.89-2.84) | 2.36 (0.87-6.45) | 1.50 (0.96-2.36) | 1.36 (0.83-2.24) |
| Intermediate | 1.01 (0.52-1.96) | 1.56 (0.79-3.08) | 1.26 (0.71-2.27) | 2.91 (0.98-8.62) | 0.81 (0.45-1.45) | 1.41 (0.83-2.38) |
| High | Ref | Ref | Ref | Ref | Ref | Ref |
| Health literacy |  |  |  |  |  |  |
| Adequate | Ref | Ref | Ref | Ref | Ref | Ref |
| Low | 0.00 (0.00-0.00) | **2.55 (1.21-5.36)** | 2.14 (0.84-5.50) | 0.96 (0.70-1.31) | **1.69 (1.13-2.53)** | 1.34 (0.86-2.10) |
| Household size (per 1 increase) | 1.23 (1.00-1.53) | 1.14 (0.98-1.32) | **1.27 (1.14-1.41)** | 1.06 (0.96-1.17) | **1.19 (1.04-1.35)** | **1.16 (1.06-1.27)** |
| Household member/steady partner with suspected infection |  |  |  |  |  |  |
| Not applicable/no | Ref | Ref | Ref | Ref | Ref | Ref |
| Yes | **3.28 (1.85-5.79)** | **2.24 (1.33-3.80)** | **2.95 (1.92-4.53)** | 0.58 (0.23-1.51) | **1.53 (1.00-2.34)** | **1.73 (1.23-2.45)** |
| **Education level** |  |  |  |  |  |  |
| Low | 0.55 (0.19-1.56) | 1.36 (0.75-2.47) | 1.38 (0.78-2.44) | 1.25 (0.70-2.21) | **1.60 (1.05-2.44)** | 1.47 (0.92-2.36) |
| Intermediate | 0.73 (0.35-1.52) | 1.06 (0.55-2.03) | 1.68 (0.99-2.86) | 1.14 (0.60-2.14) | 0.94 (0.58-1.54) | 1.29 (0.81-2.04) |
| High | Ref | Ref | Ref | Ref | Ref | Ref |
| **Susceptibility** **variables** |  |  |  |  |  |  |
| Vitamin D intake |  |  |  |  |  |  |
| Yes | Ref | Ref | Ref | Ref | Ref | Ref |
| No | 1.06 (0.60-1.87) | 1.24 (0.79-1.94) | 0.80 (0.52-1.22) | 0.76 (0.57-1.01) | 0.84 (0.60-1.18) | 0.95 (0.68-1.31) |
| BMI (per 1 increase) | 1.04 (0.99-1.10) | 1.04 (1.00-1.08) | 1.02 (0.99-1.06) | 1.01 (0.98-1.04) | 1.02 (0.99-1.05) | 1.01 (0.98-1.04) |
| Systolic blood pressure (per 1 increase) | 1.01 (0.99-1.03) | 1.00 (0.98-1.01) | 1.00 (0.99-1.01) | 1.00 (0.99-1.00) | 1.01 (0.99-1.01) | 1.00 (0.99-1.01) |
| Haemoglobin (per 1 increase) | 0.92 (0.61-1.39) | 0.89 (0.67-1.17) | 0.88 (0.69-1.10) | 0.99 (0.84-1.18) | 1.15 (0.89-1.48) | 1.02 (0.84-1.25) |
| Number of comorbidities |  |  |  |  |  |  |
| 0 comorbidities | Ref | Ref | Ref | Ref | Ref | Ref |
| 1 comorbidity | 1.11 (0.56-2.18) | 0.59 (0.33-1.06) | 0.63 (0.37-1.05) | 0.88 (0.63-1.23) | 0.96 (0.63-1.45) | 1.14 (0.76-1.73) |
| ≥2 comorbidities | 1.38 (0.57-3.35) | 1.03 (0.59-1.80) | 1.04 (0.58-1.88) | 0.87 (0.58-1.31) | 0.88 (0.49-1.58) | 1.16 (0.67-2.02) |

All models were adjusted for age (continuous) and sex.

Statistically significant p-values (p<0.05) are marked in bold.

**Table 7.** Associations between age group and presence of SARS-CoV-2 antibodies (prevalence ratios), with adjustment for individual comorbidities, per ethnic group

|  | **Prevalence ratios (95% CI) for SARS-CoV-2 infection** | | | | | |
| --- | --- | --- | --- | --- | --- | --- |
|  | **Dutch (n=464)** | **South-Asian Surinamese (n=391)** | **African Surinamese (n=360)** | **Ghanaian (n=213)** | **Turkish (n=324)** | **Moroccan (n=312)** |
| Model 1^a^ |  |  |  |  |  |  |
| Younger | 2.55 (0.93-6.97) | 0.79 (0.26-2.40) | 1.30 (0.49-3.47) | 0.57 (0.27-1.24) | 1.40 (0.60-3.30) | 1.27 (0.61-2.65) |
| Middle-aged | Ref | Ref | Ref | Ref | Ref | Ref |
| Older | 0.53 (0.16-1.74) | 0.82 (0.40-1.68) | 0.78 (0.36-1.69) | 0.95 (0.56-1.60) | 1.53 (0.72-3.26) | 0.89 (0.46-1.70) |
| Model 1^b^ + hypertension |  |  |  |  |  |  |
| Younger | **2.85 (1.01-8.06)** | 0.95 (0.31-2.96) | 1.45 (0.50-4.19) | 0.55 (0.24-1.25) | 1.22 (0.49-3.02) | 1.42 (0.66-3.04) |
| Middle-aged | Ref | Ref | Ref | Ref | Ref | Ref |
| Older | 0.42 (0.12-1.53) | 0.71 (0.35-1.47) | 0.89 (0.41-1.96) | 0.88 (0.52-1.49) | 1.64 (0.74-3.63) | 0.84 (0.42-1.65) |
| Model 1^b^ + prior CVD |  |  |  |  |  |  |
| Younger | **2.80 (1.00-7.85)** | 0.88 (0.28-2.75) | 1.51 (0.53-4.35) | 0.55 (0.24-1.28) | 1.20 (0.49-2.95) | 1.41 (0.66-3.01) |
| Middle-aged | Ref | Ref | Ref | Ref | Ref | Ref |
| Older | 0.41 (0.11-1.50) | 0.73 (0.36-1.49) | 0.86 (0.39-1.88) | 0.87 (0.51-1.48) | 1.69 (0.77-3.75) | 0.85 (0.43-1.67) |
| Model 1^b^ + diabetes |  |  |  |  |  |  |
| Younger | **2.81 (1.01-7.81)** | 0.88 (0.29-2.72) | 1.50 (0.52-4.35) | 0.55 (0.24-1.26) | 1.23 (0.50-2.99) | 1.46 (0.68-3.14) |
| Middle-aged | Ref | Ref | Ref | Ref | Ref | Ref |
| Older | 0.41 (0.11-1.48) | 0.73 (0.36-1.50) | 0.84 (0.39-1.82) | 0.88 (0.52-1.49) | 1.63 (0.73-3.61) | 0.87 (0.45-1.71) |
| Model 1^b^ + CKD risk |  |  |  |  |  |  |
| Younger | 2.71 (0.71-10.36) | 0.77 (0.25-2.40) | 1.71 (0.57-5.16) | 0.50 (0.20-1.21) | 2.08 (0.69-6.30) | 2.24 (0.88-5.75) |
| Middle-aged | Ref | Ref | Ref | Ref | Ref | Ref |
| Older | 0.40 (0.09-1.67) | 1.07 (0.48-2.37) | 0.76 (0.33-1.74) | 0.90 (0.54-1.51) | 1.60 (0.63-4.09) | 0.75 (0.34-1.66) |
| Model 1^b^ + asthma/COPD |  |  |  |  |  |  |
| Younger | **2.83 (1.02-7.84)** | 0.90 (0.29-2.78) | 1.56 (0.54-4.48) | 0.54 (0.23-1.25) | 1.34 (0.54-3.32) | 1.41 (0.66-3.02) |
| Middle-aged | Ref | Ref | Ref | Ref | Ref | Ref |
| Older | 0.41 (0.11-1.47) | 0.72 (0.35-1.48) | 0.89 (0.41-1.93) | 0.88 (0.53-1.47) | 1.50 (0.68-3.33) | 0.85 (0.43-1.67) |

Younger: <40 years; middle-aged: 40-54 years; older: ≥55 years.

Statistically significant p-values (p<0.05) are marked in bold.

^a^ Model 1 adjustments: age, sex, exposure variables (job setting, occupation level, health literacy, household size, household member with suspected infection). Adjustments for exposure variables differ per ethnic group:

- Dutch: all, but health literacy;
- South-Asian Surinamese: all, but household member with suspected infection;
- Ghanaian: all, but job setting and household size;
- African Surinamese, Turkish, Moroccan: all.

^b^ Additionally adjusted for education level.
