# Supplementary material for: Differences in SARS-CoV-2 antibody prevalence at the end of the pre-vaccination period between age groups: A cross-sectional analysis of the multi-ethnic population-based HELIUS study
Source: PLoS One. 2024 Oct 8;19(10):e0311196. doi: 10.1371/journal.pone.0311196 (PMC11460694; doi:10.1371/journal.pone.0311196)
Supplement: Attachment — Submitted filename: 2024-08-29 Response to reviewers.docx [file pone.0311196.s002.docx]

Dear Editor,

We thank all reviewers for their valuable comments on our manuscript. Below, we respond to each of the issues raised by the reviewers. Each comment is numbered and a reference is given to the corresponding part of the paper that has been revised. Please note that the page and line numbers correspond to those in the tracked changes version.

Kind regards,

Henrike Galenkamp

**Journal requirements:**

When submitting your revision, we need you to address these additional requirements.

1. Please ensure that your manuscript meets PLOS ONE's style requirements, including those for file naming. The PLOS ONE style templates can be found at

https://journals.plos.org/plosone/s/file?id=wjVg/PLOSOne_formatting_sample_main_body.pdf and

https://journals.plos.org/plosone/s/file?id=ba62/PLOSOne_formatting_sample_title_authors_affiliations.pdf

REPLY: As requested by the Editor, our revision of the manuscript now meets the style requirements.

2. Thank you for stating the following financial disclosure:

“National Institute for Public Health and the Environment (RIVM) as part of the COVID-19 research programme commissioned by the Ministry of Health, Welfare and Sports.”

Please state what role the funders took in the study. If the funders had no role, please state: "The funders had no role in study design, data collection and analysis, decision to publish, or preparation of the manuscript."

If this statement is not correct you must amend it as needed.

Please include this amended Role of Funder statement in your cover letter; we will change the online submission form on your behalf.

REPLY: We have now added this sentence to the cover letter: “*The funders had no role in study design, data collection and analysis, decision to publish, or preparation of the manuscript”.* This sentence was already part of the methods section (page 32, line 537).

3. For studies involving third-party data, we encourage authors to share any data specific to their analyses that they can legally distribute. PLOS recognizes, however, that authors may be using third-party data they do not have the rights to share. When third-party data cannot be publicly shared, authors must provide all information necessary for interested researchers to apply to gain access to the data. (https://journals.plos.org/plosone/s/data-availability#loc-acceptable-data-access-restrictions)

For any third-party data that the authors cannot legally distribute, they should include the following information in their Data Availability Statement upon submission:

1) A description of the data set and the third-party source

2) If applicable, verification of permission to use the data set

3) Confirmation of whether the authors received any special privileges in accessing the data that other researchers would not have

4) All necessary contact information others would need to apply to gain access to the data.

4. Please include your full ethics statement in the ‘Methods’ section of your manuscript file. In your statement, please include the full name of the IRB or ethics committee who approved or waived your study, as well as whether or not you obtained informed written or verbal consent. If consent was waived for your study, please include this information in your statement as well.

REPLY: The requested information is included in the manuscript (methods section P. 9, starting from line 184; and data availability statement P. 32/33).

**Reviewers' comments:**

**Reviewer #1:**

a. The risk of SARS-CoV-2 infection will always be higher in most ethnic minority groups across the globe because of their economic status and their exposure risk at work place. Therefore, I think that the exposure risk and disease risk should be assessed and compared only with variables such as economic status, outdoor activities, work environment, availability of PPEs etc. not with ethnicity.

REPLY: We agree with the reviewer that increased exposure (e.g. at work or in the household) because of lower socioeconomic status, is a hypothesized explanation for a higher risk in ethnic minority groups, which was also found in previous studies within the HELIUS cohort^1 2^. Nevertheless, the focus of this research article was the difference between age groups, and studying this in a multi-ethnic study sample has shed light on whether age differences were more apparent in certain ethnic groups independent of these factors.

b. The age wise comparison and interpretation is also not very sound in the context of people with <40 yrs are in productive age and are part of workforce in nay nation, therefore as this group is more active and defiant of various controls imposed, they end up with more exposure than older people with less activity and more comfortable with rules and controls. Authors need to revisit their findings in light of this.

REPLY: We do agree with the reviewer that the younger age group is generally more exposed, as compared to older people. In fact, this context was one of the reasons why we chose to focus on age-related prevalence of SARS-CoV-2 infection, and was part of our hypothesis. We also explored to what extent differences between age groups were explained by differences in factors that increase the exposure to the virus and whether factors related to susceptibility played a role in these associations.

On page 28, line 441 and further, we discuss the limitations of our exposure measures. On page 30, starting from line 477, we discuss our findings with regard to the exposure factors that we have examined.

c. All the interpretations of the study must be done after adjustment for confounders and explanatory factors of SARS-Cov-2 exposure and disease.

REPLY: Our aim was to study age differences, and to examine which factors explain potential differences between age groups. As such, we took a two-step approach, where we draw conclusions from the comparison of analyses that were unadjusted (only for age and sex), with analyses that were adjusted for explanatory factors. We discuss both the (absence of) differences between age groups, as well as these differences adjusted for exposure factors and susceptibility factors. It is through comparing these models that we obtain insight into how exposure and susceptibility factors affect the relationship between age and SARS-CoV-2 prevalence.

d. All the advanced serological assays which have been designed, validated, and tested for SARS-Cov-2 are based on accurate quantitation of the humoral response post natural infection.

REPLY: We assume that the reviewer approves of the methodology used.

e. Significant increase in antibody titer and epitope coverage occurs with increasing age which is an established fact in both mild and severe disease.

REPLY: Indeed, it has been demonstrated that there is a moderate positive correlation between SARS-CoV-2 IgG levels and age in adults (e.g.^3^). This is likely to be true for all ethnic groups. In addition, in our analyses we adjusted for age in years to account for linear trends within age groups (see our method section). Hence, an association between age and titers does not change our conclusions on age group differences in seroprevalence across ethnic groups.

Please refer the following articles:

1) Martin CA, Pan D, Melbourne C, Teece L, Aujayeb A, Baggaley RF, Bryant L, Carr S, Gregary B, Gupta A, Guyatt AL, John C, McManus IC, Nazareth J, Nellums LB, Reza R, Simpson S, Tobin MD, Woolf K, Zingwe S, Khunti K, Abrams KR, Gray LJ, Pareek M; UK-REACH Study Collaborative Group. Risk factors associated with SARS-CoV-2 infection in a multiethnic cohort of United Kingdom healthcare workers (UK-REACH): A cross-sectional analysis. PLoS Med. 2022 May 26;19(5):e1004015. doi: 10.1371/journal.pmed.1004015. PMID: 35617423; PMCID: PMC9187071.

2) Patel M, Nair M, Pirozzoli E, Cienfuegos MC, Aitken E. Prevalence and socio-demographic factors of SARS-CoV-2 antibody in multi-ethnic healthcare workers. Clin Med (Lond). 2021 Jan;21(1):e5-e8. doi: 10.7861/clinmed.2020-0619. PMID: 33479076; PMCID: PMC7850215.

3) Martin CA, Woolf K, Bryant L, Goss C, Gogoi M, Lagrata S, Papineni P, Qureshi I, Wobi F, Nellums L, Khunti K, Pareek M; UK-REACH Study Collaborative Group. Coverage, completion and outcomes of COVID-19 risk assessments in a multi-ethnic nationwide cohort of UK healthcare workers: a cross-sectional analysis from the UK-REACH Study. Occup Environ Med. 2023 Jul;80(7):399-406. doi: 10.1136/oemed-2022-108700. Epub 2023 May 23. PMID: 37221040; PMCID: PMC10314065.

4) Siddiq S, Ahmed S, Akram I. Clinical outcomes following COVID-19 infection in ethnic minority groups in the UK: a systematic review and meta-analysis. Public Health. 2023 Sep;222:205-214. doi: 10.1016/j.puhe.2022.05.019. Epub 2022 Jun 9. PMID: 35970621; PMCID: PMC9181265.

5) Irizar P, Pan D, Kapadia D, Bécares L, Sze S, Taylor H, Amele S, Kibuchi E, Divall P, Gray LJ, Nellums LB, Katikireddi SV, Pareek M. Ethnic inequalities in COVID-19 infection, hospitalisation, intensive care admission, and death: a global systematic review and meta-analysis of over 200 million study participants. EClinicalMedicine. 2023 Mar;57:101877. doi: 10.1016/j.eclinm.2023.101877. Epub 2023 Mar 6. PMID: 36969795; PMCID: PMC9986034.

6) Agyemang C, Richters A, Jolani S, Hendriks S, Zalpuri S, Yu E, Pijls B, Prins M, Stronks K, Zeegers MP. Ethnic minority status as social determinant for COVID-19 infection, hospitalisation, severity, ICU admission and deaths in the early phase of the pandemic: a meta-analysis. BMJ Glob Health. 2021 Nov;6(11):e007433. doi: 10.1136/bmjgh-2021-007433. PMID: 34740916; PMCID: PMC8573300.

REPLY: We thank the reviewer for providing us with these relevant references. We have added the references to the following systematic reviews/meta-analyses (page 6, line 112), which focus on the general population akin to our study (and not solely on health care workers):

-Siddiq S, Ahmed S, Akram I. Clinical outcomes following COVID-19 infection in ethnic minority groups in the UK: a systematic review and meta-analysis. Public Health. 2023 Sep;222:205-214. doi: 10.1016/j.puhe.2022.05.019. Epub 2022 Jun 9. PMID: 35970621; PMCID: PMC9181265.

-Irizar P, Pan D, Kapadia D, Bécares L, Sze S, Taylor H, Amele S, Kibuchi E, Divall P, Gray LJ, Nellums LB, Katikireddi SV, Pareek M. Ethnic inequalities in COVID-19 infection, hospitalisation, intensive care admission, and death: a global systematic review and meta-analysis of over 200 million study participants. EClinicalMedicine. 2023 Mar;57:101877. doi: 10.1016/j.eclinm.2023.101877. Epub 2023 Mar 6. PMID: 36969795; PMCID: PMC9986034.

-Agyemang C, Richters A, Jolani S, Hendriks S, Zalpuri S, Yu E, Pijls B, Prins M, Stronks K, Zeegers MP. Ethnic minority status as social determinant for COVID-19 infection, hospitalisation, severity, ICU admission and deaths in the early phase of the pandemic: a meta-analysis. BMJ Glob Health. 2021 Nov;6(11):e007433. doi: 10.1136/bmjgh-2021-007433. PMID: 34740916; PMCID: PMC8573300.

Reviewer #2:

In the work "Differences in SARS-CoV-2 antibody prevalence at the end of the pre-vaccination period between age groups : a cross-sectional analysis of the multi-ethnic population- based HELIUS study" authors investigated SARS-CoV-2 seroprevalence and did not find statistically significant differences across age groups. Particular focus of their study is ethnic component and investigation of the age differences for seroprevalence.

I found the study interesting and important for the field. The core minus is that the data is out of hot topic and belong to the first part of the COVID-19 pandemic.

However, I have few major comments which I believe should be satisfied in order to make the manuscript accepted for publication.

1. It would be good if authors remake the figures to better point differences not only for age, but also across ethnic groups. Possibly putting them on one plot or choosing any other way that data for age, ethnicity can be on the same plot to allow quantitative comparison between groups

REPLY: We agree with the reviewer on the usefulness of a figure that includes both age differences and ethnic differences in the probability of infection. We refer to the already included Figure 3 (which was Figure 2 in the original submission), and the newly added Figure 2, in which age differences are depicted within each ethnic group and in which age group differences can be compared across ethnic groups. Figure 3 shows 1) that age group differences were statistically significant in none of the ethnic groups, and 2) that prevalence ratios tended to be larger in the Dutch group, as compared to the other groups, even though they were not statistically significant.

2. I recommend to complement the manuscript with more quantitative information in order to make the text and data maximally useful for the epidemiology community.

REPLY: We added quantified results to the text of the manuscript:

*page 17, line 342:*

*After adjustments for age and sex (model 0), there were no statistically significant age group differences in SARS-CoV-2 seroprevalence in the six ethnic groups (Figs 2 and 3, Table 3). The direction of estimates for the association of age group with SARS-CoV-2 seroprevalence varied by ethnic group. For instance, PRs pointed towards a higher seroprevalence among younger adults (<40 years) compared to middle-aged adults (40-54 years) in the Dutch (PR=2.01, 95% CI 0.75-5.36), African Surinamese (PR=1.25, 95% CI 0.54-2.91), Turkish (PR=1.43, 95% CI 0.70-2.90) and Moroccan groups (PR=1.08, 95% CI 0.58-2.03), but towards a lower seroprevalence in younger adults in the South-Asian Surinamese (PR=0.89, 95% CI 0.33-2.43) and Ghanaian groups (PR=0.73, 95% CI 0.36-1.51). Age group differences in seroprevalence were most pronounced in the participants of Dutch origin.*

*page 21, line 365:*

*Some exposure variables, particularly health literacy, household size, household member/steady partner with suspected infection, were significantly associated with SARS-CoV-2 seroprevalence (Supplemental Table S4). Additional adjustments for these variables resulted in changes of estimates for the effect of age group (model 1), mainly towards larger differences between age groups (Fig 2, Table 3). However, the patterns of changes were not consistent across ethnic groups. For instance, in some ethnic groups, these adjustments mainly led to a higher PR in the younger group versus middle-aged group (e.g., Dutch: PR changed from 2.01 to 2.55, and African Surinamese: from 1.25 to 1.30) but to little change in PR for the older group. The reverse was observed in other groups, such as the Turkish group (PR for the younger group compared to the middle-aged group changed from 1.43 to 1.40).*

3. Better elaboration of the current data in terms of seroconversion, maybe comparison with other infectious diseases would make the manuscript much better and useful for scientific community. Because the topic authors explore in the manuscript is old, it is necessary to present information in the context of the current epidemic status and possible future dangers

REPLY: We added the following to the discussion section:

*Page 30, line 508: Thus, for the future out roll of targeted prevention activities in outbreaks of COVID-19 and other infections with a major impact on the health of older individuals, it is important to realise that older adults in ethnic minority groups may be at an increased risk. This will be especially relevant if it would turn out that, due to lower vaccine uptake, COVID-19 vaccination has not been able to eliminate, or at least diminish, this higher susceptibility.*

Regarding the other points raised by the reviewer: If we use HELIUS data from the more recent waves involving the omicron variant, almost all participants had anti-SARS-CoV-2 antibodies (the majority of which was from having infection) regardless of ethnic group. If we use influenza as an example of another infectious disease, its spread is mostly driven by children between the ages of 5-11). Making any inference of this epidemic on other epidemics would not be suited given the different epidemiological context (information on the context was give on page 6/7, lines 130-137) .

4. It would be great to see comparison with other studies, especially in Asia. Moreover, would be great to compare results for the ethnic groups in the Netherlands and their home countries.

REPLY: The current paper focused on age differences in a multi-ethnic population, and not on ethnic differences or age differences per se. Other studies that have looked at age differences in SARS-CoV-2 antibody prevalence across ethnic groups were not found, unfortunately. In addition, we are not aware of studies in home countries (Turkey, Morocco etc.) with which comparisons could be made.

5. I would recommend statistical information in the tables transfer to the visual from, possibly keeping tables for the supplementary part.

REPLY: Agreed. We have omitted Table 2 from the manuscript, and added an extra Figure (Figure 1, page 17) to depict the unadjusted differences in SARS-CoV-2 antibody test result.

In addition, we omitted Table 4, as information in this Table was overlapping with that in Figure 2a-2f.

Tables 2 and 4 were transferred to the supplementary part (Tables S4 and S5, respectively).

6. Is it possible to provide also geospatial characteristics of the seroconversion? If the data are available, certain local differences even within the same ethnic group can be interesting, especially in the context of pandemic management considering local peculiarities. The Dutch group is the largest and spread over the whole country, but what about other ethnic groups? Do we see differences between small towns and large cities?

REPLY: The HELIUS study is conducted in Amsterdam, and as such it was not possible to examine differences between towns or cities. However, previous work in HELIUS, and in Amsterdam have indeed shown independent associations between infections and city districts and migration background.^1 4^

REPLY: We have added this to page 31, line 502:

*“It was indeed found that, next to migration background, infection rates in Amsterdam were also independently associated with lower SES city districts (Bachour et al.).”*

7. In general, I would recommend more focus on the visual representation of the data in order to present the conclusions in the best possible way for the reader.

REPLY: We refer the Reviewer to our response to comment 5.

1. Coyer L, Boyd A, Schinkel J, et al. Differences in SARS-CoV-2 infections during the first and second wave of SARS-CoV-2 between six ethnic groups in Amsterdam, the Netherlands: A population-based longitudinal serological study. *Lancet Reg Health Eur* 2022;13:100284. doi: 10.1016/j.lanepe.2021.100284 [published Online First: 20211214]

2. Coyer L, Boyd A, Schinkel J, et al. SARS-CoV-2 antibody prevalence and correlates of six ethnic groups living in Amsterdam, the Netherlands: a population-based cross-sectional study, June–October 2020. *BMJ open* 2022;12(1):e052752 %@ 2044-6055.

3. Zhai B, Clarke K, Bauer DL, et al. SARS-CoV-2 antibody response is associated with age and body mass index in convalescent outpatients. *The Journal of Immunology* 2022;208(7):1711-18.

4. Bachour Y, Wynberg E, Coyer L, et al. COVID-19 burden differed by city districts and ethnicities during the pre-vaccination era in Amsterdam, the Netherlands. *Front Public Health* 2023;11:1166193. doi: 10.3389/fpubh.2023.1166193 [published Online First: 20230623]
